# Myeloid Cell Diversity and Impact of Metabolic Cues during Atherosclerosis

**DOI:** 10.20900/immunometab20200028

**Published:** 2020-10

**Authors:** Alexandre Gallerand, Marion I. Stunault, Johanna Merlin, Rodolphe R. Guinamard, Laurent Yvan-Charvet, Stoyan Ivanov

**Affiliations:** Mediterranean center of molecular medicine (C3M)-Université Côte d’Azur–INSERM U1065, Team 13, Nice, 06200, France

**Keywords:** macrophage, monocyte, dendritic cell, metabolism, atherosclerosis

## Abstract

Myeloid cells are key contributors to tissue, immune and metabolic homeostasis and their alteration fuels inflammation and associated disorders such as atherosclerosis. Conversely, in a classical chicken-and-egg situation, systemic and local metabolism, together with receptor-mediated activation, regulate intracellular metabolism and reprogram myeloid cell functions. Those regulatory loops are notable during the development of atherosclerotic lesions. Therefore, understanding the intricate metabolic mechanisms regulating myeloid cell biology could lead to innovative approaches to prevent and treat cardiovascular diseases. In this review, we will attempt to summarize the different metabolic factors regulating myeloid cell homeostasis and contribution to atherosclerosis, the most frequent cardiovascular disease.

## Introduction

Atherosclerosis is a major vascular disease that continuously spreads worldwide. Atherosclerosis contributes to cardiovascular disease (CVD)-related deaths, estimated to account for more than 17 million deaths per year worldwide, making this pathology a major public health issue. Atherosclerosis is described as a metabolic disease associated with a chronic low-grade inflammation linked to lipid accumulation in the intima of large and medium-sized arteries, which favors plaque formation [1,2]. Since the 1950’s, mounting evidence linked cholesterol metabolism to atherosclerosis development. Atherosclerotic patients not only showed increased serum cholesterol levels, and more specifically cholesterol present in the low-density lipoproteins fraction (LDL), but also accumulation of cholesterol in macrophage foam cells pointing out to cholesterol as a culprit of immunometabolic perturbations in the establishment and development of atherosclerosis. LDL-cholesterol levels are now considered as an independent risk factor for CVDs [3,4]. Interestingly, LDL accumulation into the arterial wall is associated with inflammatory signals which trigger the attraction of myeloid cells such as dendritic cells (DC), neutrophils, macrophages and monocytes [5]. Advanced atherosclerotic plaques are complex structures containing lipids, necrotic cores, calcification zones and immune cells [6]. Plaque growth increases arterial stiffness and could be responsible for disturbed blood flow, while their rupture can lead to ischaemic strokes and transient cerebral ischaemic attacks [7].

During the past two decades, tremendous progress has been made highlighting the involvement of immune cells at all stages of the disease including plaque initiation, development and rupture. Particularly, the accumulation of myeloid cells in human atheromatous plaques is a strong marker of plaque instability and predictor of negative outcome [8,9]. The respective role of the different myeloid cell types in the establishment and progression of the disease has since been thoroughly investigated using pre-clinical mouse models. Monocytes, which enter atheromatous plaques from the blood circulation and differentiate into macrophages, are the main culprits of atherosclerosis development. Further mechanistic complexity came later with the realization of the involvement of neutrophils and DCs in the disease, the latter driving and bringing adaptative immunity in the picture.

Myeloid cell precursors have diverse origins: some emerge from hematopoiesis in the bone marrow, while others arise from primitive embryonic structures [10]. Their functional diversity is thought to be acquired via the action of tissue-specific cues but little is known on how this local developmental imprinting of myeloid cells takes place [11]. This observation particularly stands regarding myeloid cell immunometabolism, as microenvironmental signals can trigger rapid metabolic adaptations to adjust the immune response. In atherosclerosis, myeloid cell metabolism influences plaque development [12]. For example, under inflammatory conditions, macrophages display increased glycolytic metabolism and Glut1 expression, the main myeloid cell glucose transporter [13]. Monocytes and macrophages from atherosclerotic patients show increased mitochondrial oxygen consumption rate (OCR). Together these findings suggest a global change in metabolic activation state [14].

A metabolomics-based analysis of human carotid plaques revealed a correlation between plaque metabolic signatures, namely elevated glycolysis and low fatty-acid oxidation, and the presence of plaque instability features [15]. This observation strongly suggests that intra-plaque metabolic cues may determine the outcome of the disease. A better understanding of how metabolites affect in situ myeloid cell activation would help for the design of new therapeutic approaches to prevent and treat atherosclerosis. In this review, we will address how metabolic signals impact on myeloid cell diversity and function in the context of atherosclerosis.

## Immune Cell Diversity in Plaques

Pioneering studies revealed that human plaques contain a variety of immune cells. These observations were repeated in mouse pre-clinical models of atherosclerosis development, namely Ldlr^−/−^ and ApoE^−/−^ mice. Although wild-type mice are protected against the disease, high cholesterol diet feeding of Ldlr^−/−^ and ApoE^−/−^ mice promotes hypercholesterolemia and atherosclerosis development. Descriptions of plaque immune cells were initially based on immunochemistry and demonstrated the presence of macrophages, B and T cells in plaque lesions [16]. Nevertheless, the limited number of parameters available was not adapted to grasp the full spectrum of immune cells residing in advanced plaques. With the improvement of flow cytometry, the number of parameters simultaneously analyzed progressively increased and a further complexity in plaque immune cell populations emerged [17,18]. Single-cell RNA-Seq and Cytometry by Time of Flight (CyTOF) technologies further extended our ability to discover and characterize new tissue-resident immune populations and their activation states. This technological leap offered a new perspective to decipher in greater depth immune cell diversity in atherosclerotic plaques [19]. In the past two years, multiple studies applied single-cell RNA-Seq techniques to human plaque samples from endarterectomy patients [20], and to aortic cells extracted from wild-type and atherosclerotic mice [18,21–25]. This generated an extensive characterization of the blood vessel-residing immune landscape in health and disease (Tables 1 and 2). Interestingly, only a small number of myeloid cells (of which around 70% were monocytes) were observed at steady state in the aortic wall of wild-type mice [25]. It seems reasonable that monocytes crawling on endothelial cells were the main population of immune cells detected in those studies. Leukocyte diversity was shown to greatly increase in the aorta during atherosclerosis development, as neutrophils, T cells, B cells and NK cells were also identified in Ldlr^−/−^and ApoE^−/−^ mice fed a chow or a high fat diet [21,22,24]. This diversity was also observed in human samples [20,22].

However, the relative proportion of each cell type reported by different single-cell studies shows significant fluctuations. In mice, macrophages were reported to represent from 9 to 80% of the aortic leukocyte pool and, inversely, T cells represented from 3 to 60% of leukocytes. The same variations were observed in human samples (Table 2). These differences could be explained by multiple factors, and notably differences in tissue digestion technique, leukocyte purification method, and the markers and transcripts used for cell type identification. The entry of atherosclerosis research in the single cell RNA-Seq era could therefore benefit from a universal experimental pipeline that would facilitate comparison between studies. A first step toward this direction could consist in generating a meta-analysis of the data from the studies summarized in Table 1 to characterize plaque composition. A meta-analysis study was recently published and evidenced the immune cell diversity in plaque and the markers allowing to define each population [26].

These studies illustrate that single-cell approaches are amongst the most powerful tools to precisely identify cell subsets as well as their respective metabolic demands. Myeloid cells were broadly shown to represent a significant part (up to 90%) of immune cells in atherosclerotic lesions [23]. Here, we will briefly discuss the identity and origin of plaque resident myeloid cells.

### Monocytes

Monocytes are short-lived mononuclear phagocytes that are generated in the bone marrow (BM) during hematopoiesis [27,28]. They rely on CSF1R (Colony Stimulating Factor 1 Receptor) signaling for their development and survival [29,30]. Among leukocytes, which have been positively correlated with cardiovascular events in humans [31–33], monocytes play a pivotal role in atherosclerosis development. Hypercholesterolemia, a key component of atherosclerosis, has been associated with increased circulating monocyte numbers (monocytosis) in mice, rabbits, swines and humans [34–36].

Circulating monocytes are identified as CD11b^+^ CD115^+^ cells, and exist as two functionally distinct subsets in both mice and humans: classical monocytes, identified as Ly6C^high^ in mice and CD14^high^ CD16^low^ in humans, and non-classical monocytes, identified as Ly6C^low^ and CD14^low^ CD16^high^ [37]. An additional human population of CD14^+^CD16^+^ monocytes has also been documented. Developmentally, Ly6C^low^ monocytes were proposed to arise from Ly6C^high^ monocytes [38,39]. Ly6C^low^ monocytes require the transcription factor Nr4a1 (Nur77) for their maturation [40], they are commonly referred to as “patrolling monocytes”, as they are closely associated to the endothelium which they survey in order to remove dead endothelial cells [41,42].

Ly6C^high^ monocytes are also called “inflammatory” monocytes, as they accumulate during infections and are preferentially recruited to inflamed tissues [38]. They display a high expression of CCR2 [43,44], the main chemokine receptor governing monocyte recruitment to inflammatory sites [45] as well as into atherosclerotic plaques. Plaque initiation is driven by Ly6C^high^ monocyte recruitment to the intima of the endothelial wall [46–50]. Their inflammatory nature seems to be supported by the fact that osteopetrotic mice, which lack functional CSF1 (Colony Stimulating Factor 1) and therefore monocytes [51], are protected against hypercholesterolemia-induced atherosclerosis [52–54]. In 1998, two independent studies documented the key role played by monocyte recruitment to atheromatous lesions through the CCL2-CCR2 chemotactic axis, as mice deficient for CCR2 [55] or its ligand CCL2 [56] also displayed reduced atherosclerotic lesions. Monocyte chemotaxis in atherosclerosis was further characterized in 2007, as Tacke and colleagues provided evidence of the relative contribution of CCR2, CCR5 and CX3CR1 for monocyte recruitment into plaques [47]. CX3CR1 is highly expressed on Ly6C^low^ monocytes and remains detectable on Ly6C^high^ monocyte subset [57]. In contrast, CCR2 is predominantly expressed by the Ly6C^high^ population [57].

Understanding the roles played by metabolism in monocyte biology is of key importance considering the limitations of current therapies. Indeed, limiting monocyte recruitment to the plaque seems to be a reasonable strategy for reducing the development of atherosclerotic lesions. Complementarily, new anti-inflammatory approaches have recently gained great interest. Indeed, lowering the inflammatory response has emerged as a novel therapeutic target to decrease CVDs related death and comorbidities. Interleukine-6 (IL-6) and IL-1β are two well-established pro-inflammatory mediators and their levels are increased during atherosclerosis progression [58]. Among those two, the pro-inflammatory cytokine IL-1β emerged as a major mediator of atherosclerosis development [59–61]. Surprisingly, IL-1β signaling was also shown to be an important component of plaque remodeling and stability [62]. The Canakinumab Anti-inflammatory Thrombosis Outcome Study (CANTOS) trial showed that patients treated with the IL-1β-targeting monoclonal antibody Canakinumab had a lowered cardiovascular-event-related mortality rate. However, Canakinumab treatment induced various side effects and increased susceptibility to infections, resulting in no overall survival benefits and underlining the urgent need for other therapeutic approaches [63,64].

### Macrophages

Macrophages are highly phagocytic cells that can be identified through their co-expression of CD64 and MerTK [11]. Macrophages are ubiquitously present across organs and play key roles both in health and disease [11]. A key function of tissue-resident macrophage is the removal of dead cells, a process named efferocytosis [65]. Every day around 0.4% of the total estimated number of 3.7 × 10^13^ cells dye in the adult human body [66]. Although all macrophage populations perform efferocytosis, they also display tissue-specific functions such as heme detoxification and iron handling in the spleen, surfactant clearing in the lungs or thermogenesis regulation in brown adipose tissue [67,68]. This functional heterogeneity can partly be explained by the developmental origin of macrophages. Microglia, the population of brain resident macrophages, arise from yolk sac precursors present at early developmental stages [69,70]. Alveolar pulmonary macrophages originate from foetal liver progenitors [71] while the population of gut macrophages derives from bone marrow precursors [72]. Consequently, embryonically-derived macrophages and monocyte-derived macrophages often coexist in adult tissues [73,74]. Like monocytes, tissue-resident macrophages rely on CSF1R signalling, which can bind either IL-34 or CSF1, for their maintenance. Interestingly, a tissue specificity for either ligand has been observed among tissue-resident macrophages, as microglia rely on IL-34 while large peritoneal macrophages rely exclusively on CSF1 [75,76]. Furthermore, macrophage heterogeneity can be attributed to local environmental features, even between subsets that share a common developmental origin [11].

**Figure 1:**
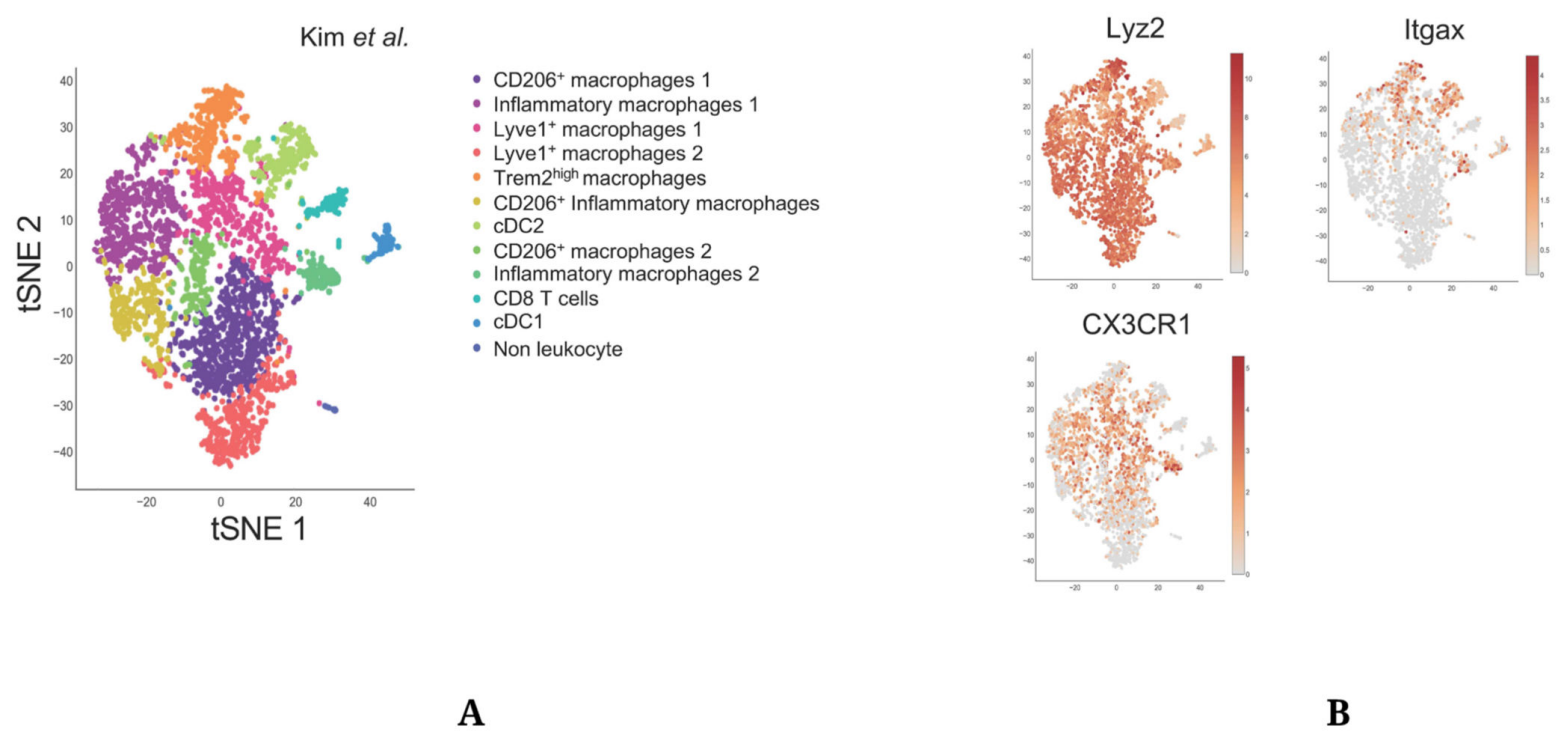
Single-Cell approaches highlight plaque immune cell diversity. (**A**) Single-Cell RNA-Seq of aortic CD45^+^ cells from Ldlr^−/−^ mice fed a HFD for 12 weeks. Data from Kim et al. [23] (GSM3215435) were analyzed using the Single-Cell Explorer software. **List of markers used**: CD206^+^ Macrophages: Fcgr1, Itgam, Mafb, Mrc1. Inflammatory macrophages: Fcgr1, Itgam, Mafb, NLRP3, IL1b, Nfkbia. Lyve1^+^ macrophages: Fcgr1, Itgam, Mafb, Lyve1. TREM2^high^ macrophages: Fcgr1, Itgam, Mafb, TREM2, ABCG1, Lpl, Lipa. CD206^+^ Inflammatory macrophages: Fcgr1, Itgam, Mafb, Mrc1, NLRP3, IL1b (low), Nfkbia, TNF. CD8 T cells: Lck, CD3, CD8. cDC2: Zbtb46, Itgax, Flt3, Itgam (+), Itgae (−). cDC1: Zbtb46, Itgax, Flt3, Itgam (−), Itgae, IRF8. Non leukocyte: Ptprpc (−). (**B**) Expression pattern of genes used for targeted Cre expression in myeloid cells.

Atheromatous plaque macrophages are monocyte-derived macrophages with the ability to proliferate in situ following their recruitment and differentiation. The understanding of macrophage diversity in metabolic disorders and inflammatory diseases is of particular interest during atherosclerosis, as multiple macrophage subsets with specific immune profiles and functions have been observed within the plaque [18,21–23] (Figure 1A). Our knowledge on plaque macrophage diversity was previously restricted to pro-inflammatory “M1” macrophages, anti-inflammatory “M2” macrophages, and foam cells which were considered inflammatory cells [5]. The in situ identification of these subsets was based on immunohistochemistry approaches, while their functions were explored using in vitro models. However, new single cell methods have now revealed more layers of complexity in plaque macrophage subsets [18,21–23]. Notably, the single-cell RNA-Seq dataset from Kim and colleagues [23], which displays the greater myeloid cell enrichment, shows the existence of several distinct populations of inflammatory and anti-inflammatory subsets. Surprisingly, expression of the archetypical anti-inflammatory macrophage marker CD206 also appears on populations expressing inflammatory markers (Figure 1A). Two macrophage populations expressed high levels of Lyve1, a marker associated with tissue-resident macrophages [77] which were also identified by Cochain et al. [21] (Figure 1A). This new technology also allowed a detailed in vivo characterization of foam cells, identified as Trem2^high^ macrophages [23,78,79].

Single cell studies now pave the way to understanding plaque macrophage biology. Further investigation is needed to determine how these different subsets participate to inflammation or its resolution via efferocytosis and plaque remodelling [19]. To establish the developmental connection between plaque macrophage populations and their particular localization and metabolic demands is of crucial significance to understand the pathological mechanisms occurring during atherosclerosis progression.

### Dendritic Cells

DCs are professional antigen presenting cells (APC). Two major DCs populations have been identified in mice and humans: the conventional (cDCs) and the plasmacytoid dendritic cells (pDCs). Both human and mouse cDCs highly and selectively express the transcription factor Zbtb46 (Zinc finger and BTB domain containing 46) [80,81]. Zbtb46 is not expressed by other myeloid cells such as macrophages, monocytes or neutrophils. In mice, cDCs populations highly express CD11c and MHC II and two main subsets have been identified in lymphoid and non-lymphoid tissues. In lymphoid tissues, cDC1s express CD8, CD24 and XCR1 while cDC2s are characterized by CD4 and Sirpα expression [82]. In the majority of nonlymphoid tissues, cDC1s are described as CD103^+^ XCR1^+^ and cDC2s as CD11b^+^ Sirpα^+^. cDC1 and cDC2 require specific transcription factors for their development. cDC1 depend on BATF3 (Basic Leucine Zipper activating transcription factor–like transcription factor 3) and IRF8 (IFN regulatory factor 8) while cDC2 rely on IRF4 and Notch2 for their development and maintenance [82]. A key feature of cDCs is their high capacity to capture antigens in peripheral tissues and subsequently migrate to local draining lymph nodes to initiate the adaptive immune response. Another major function of cDCs is the production of pro-inflammatory cytokine such as IL-6, TNFα and IL-1 following activation of innate immunity receptors. This cytokine production leads to immune cell recruitment and mobilization and allows for specific and efficient immune responses. On the other hand, pDCs essentially release type 1 interferons (IFN-I), both IFNα and IFNβ, in response to virus infections [83]. Their potential implication in atherosclerosis is suggested by the fact that IFN-I decreases macrophage phagocytic abilities [84] and that IFNAR-deficient animals have decreased plaque area and macrophage content [85].

In the context of atherosclerosis, cDCs contribute to chronic inflammation by attracting and activating T cells [86]. The production of CCL17 by mature cDCs contributes to CD4^+^ T cells and regulatory T cells (Tregs) migration and recruitment to plaques. CCL17 deletion leads to a slower atherosclerosis progression and a decreased number of macrophages and T cells in plaques [87]. The presence of CD4^+^ T cells with a phenotype of antigen activated (CD44^+^) cells was documented in mouse atherosclerotic models [17]. CD4^+^ T cells stimulation requires a peptide loading on major histocompatibility complex (MHC II), selectively expressed by antigen presenting cells. The cDC antigen presentation function seems to play a pivotal role in the progression of atherosclerosis. Nevertheless, and despite recent progress in the field, the nature of the antigen (peptide or lipid) remains to be fully understood. For instance, ApoB (the core protein in LDL) reactive CD4^+^ T cells were identified in pre-clinical atherosclerotic models [17] and humans [88]. Immunization strategies were developed using ApoB epitopes and those demonstrated atheroprotective effect, illustrated by reduced plaque area, when conjugated to appropriate adjuvants [89,90]. This protection was associated with increased IL-10 production, an anti-inflammatory cytokine mainly secreted by regulatory T cells (Tregs). In atherosclerotic patients, an oligoclonal T cell repertoire was observed in comparison to healthy patients [91,92]. This observation further supports the relevance of antigen presentation during disease development. Recently, the generation of MHC II tetramers loaded with ApoB-derived peptide revealed that the majority of ApoB-recognizing T cells are T regs [88]. Moreover, the deletion of two important costimulatory molecules: CD80 and CD86 in mice DCs decreased T-cell activation/infiltration in plaques [93] demonstrating that cDCs play a crucial role during disease development.

In advanced plaques, apoptotic cell accumulation due to defective efferocytosis leads to DNGR-1 activation (dendritic cell NK lectin group receptor-1) on CD8a^+^ cDC1s, which blunts IL-10 production, therefore contributing to atherosclerosis aggravation [94]. However, the mechanisms underlying the defective efferocytosis in DCs are still unknown and need to be deciphered. In conclusion, DCs, as pivotal players linking innate and adaptive immunity, offer new insights that may lead to new therapeutic targets and notably vaccination strategies.

### Neutrophils

Neutrophils are associated with the early inflammatory response [95]. Neutrophils have been shown to either be able to directly affect atherogenesis [96], or contribute to pathology onset by driving immune cell entry in atherosclerotic lesions [97] and by promoting plaque rupture [98] respectively. This suggests an important crosstalk between neutrophils and other immune and stromal cells.

Growing evidence suggests that neutrophils play a pivotal role in the initiation of atherosclerosis. Neutrophil adhesion to the endothelial wall through CCL3 and CCL5 binding on CCR1, CCR3 or CCR5 triggers neutrophils extravasation and their entry into plaques [99]. There, activated neutrophils release granule proteins containing chemotactic “alarmins”, such as cathelicidin/LL-37 in Human (CRAMP in mice), Human α-defensins (human neutrophil peptides, HNPs), azurocidin (HBP, CAP37) and serprocidins (elastase, cathepsin G, proteinase-3), inducing leukocytes attraction and recruitment to the site of inflammation (for review see [100]). In addition, S100A8/A9, a cytoplasmic protein, reduces neutrophils rolling on the endothelial wall and activates β2 integrin to facilitate leukocyte extravasation and entry to the site of inflammation [101]. Interestingly, alarmins have also been reported to contribute to the activation of inflammasomes such as NLRP3 [102]. NLRP3 activation leads to IL-1β and IL-18 production and to the HMGB1 alarmin (High-mobility group box 1 protein) release, creating a loop that amplifies innate immune responses [103]. NLRP3 inflammasome activation then increases neutrophil recruitment to inflammatory sites leading to the activation of neutrophil extracellular traps (NETs) [104]. NETs are web-like fiber structures released by neutrophils and made of extracellular chromatin, nuclear proteins, and serine proteases. NETs are known to increase monocyte recruitment to inflamed sites and trigger reactive oxygen species (ROS) and proinflammatory cytokines release by macrophages [105,106]. In this context, NETs may promote type I interferon (IFN-I) release from pDCs contributing therefore to atherosclerosis progression and suggesting an essential crosstalk between neutrophils and pDCs [107].

Neutrophils have been found at sites of plaque rupture in patients with acute coronary syndrome [108]. Interestingly, neutrophils are essentially located in the unstable layers of human atherosclerotic lesions with a high inflammatory activity and also correlated to the elevated numbers of monocytes found in these regions [99]. In addition, NETs are thought to be involved in plaque destabilization through the induction of endothelial cell wall cytotoxicity in humans [109,110]. Neutrophils are the main producer of myeloperoxidase (MPO) [111]. MPO is a heme-containing peroxidase that catalyzes the formation of reactive oxygen species intermediates [112] that induce macrophage cholesterylester accumulation and foam cell formation, leading to atherosclerosis aggravation [113]. Recent studies have highlighted that neutrophils undergo transcriptional regulations under inflammatory conditions and NETosis [114,115]. The significance of NETs during atherosclerosis was extensively described in a recent manuscript [115].

### Mouse Models

Mouse Cre-Lox systems have extensively been used to explore the role of myeloid cell functions in atherosclerosis. Lyz2^Cre^, CX3CR1^Cre^ and CD11c^Cre^ mice were the most commonly used to study macrophages and neutrophils, monocytes, and dendritic cells respectively. Although these genes are dominantly expressed by the aforementioned cell types, some well-documented overlaps in their expression exist between myeloid cell types. Single-cell sequencing approaches have now brought to light the subset-specific expression pattern of these genes within the plaque (Figure 1B), which may allow more specific targeting of myeloid subsets within plaques and reinterpretation of previously generated data.

As expected, Lyz2^Cre^ appears to be virtually ubiquitously expressed across plaque resident myeloid cells. Although CX3CR1 expression is only restricted to certain myeloid subsets within the plaque, most macrophages are monocyte-derived cells which therefore expressed CX3CR1-driven Cre at an earlier differentiation stage. However, the use of inducible CX3CR1^CreERT2^ models gives more flexibility to the model. As an example, Lin and colleagues recently used Cx3cr1^CreERT2-IRES-YFP/+^Rosa26^fl-tdTomato/+^ mice in a fate-mapping and single-cell approach [18]. The authors induced Cre expression when plaques were established, immediately prior to plaque regression induction in order to differentially characterize CX3CR1^+^ plaque cells and cells derived from CX3CR1^+^ precursors [18]. CD11c^Cre^ mice were extensively used to characterize DCs functions in health and disease. However, CD11c was also shown to be expressed by Ly6C^low^ monocytes [57] which can, to a lower extent than Ly6C^high^ monocytes, infiltrate atheromatous lesions [47]. As shown in Figure 1B, plaque expression of CD11c is not restricted to DCs, but also concerns certain macrophage subsets including the now well identified TREM2^high^ foam cells [23,78,79]. CD11c^Cre^ mice could therefore be a valuable model to study foam cell metabolism during atherosclerosis.

## Metabolic Phenotype of Plaque Immune Cells

Atherosclerosis progression is accompanied by a modulation of systemic and plaque metabolites. Recently, non-invasive imaging techniques, commonly used in oncology, were deployed to predict rupture-prone plaques. Positron emission tomography (PET) is traditionally employed to investigate myocardial reperfusion. PET/CT studies revealed an accumulation of the glucose analog 18F-fluoro-2-deoxy-d-glucose (18F-FDG) in atherosclerotic lesions in humans [116]. This suggested increased glucose avidity and potentially metabolization in plaque residing cells. A metabolomic analysis performed on iliac-femoral arteries extracted from control and atherogenic rabbits revealed an increased abundance of glycolysis and pentose phosphate pathway (PPP) metabolites in plaque-enriched vessels [117]. Whether these metabolites accumulate in specific immune or stromal cell-type remains to be established. In the following section we will discuss the impact of myeloid cell glucose metabolism on plaque development.

### Lipid Handling

#### Monocytes

Hypercholesterolemia, the predominant metabolic feature of cardiovascular diseases, is known to influence hematopoiesis and induce a differentiation bias of hematopoietic stem cells (HSCs) towards the myeloid lineage. Indeed, HSCs obtained from ABCA1^−/−^ ABCG1^−/−^ mice, lacking transporters involved in cholesterol efflux, display increased proliferation and myelopoiesis switch [118]. This phenomenon is amplified in ApoE^−/−^ mice, one of the most commonly used murine models of hypercholesterolemia-induced atherosclerosis [119]. Taken together, recent data on cholesterol-related myelopoiesis exacerbation suggest that an increase in cellular cholesterol content promotes membrane lipid raft formation in HSCs, thus promoting the stabilization of chemokine and cytokine receptors at the cell surface and signal HSCs to quit quiescence [118,120–122].

**Figure 2:**
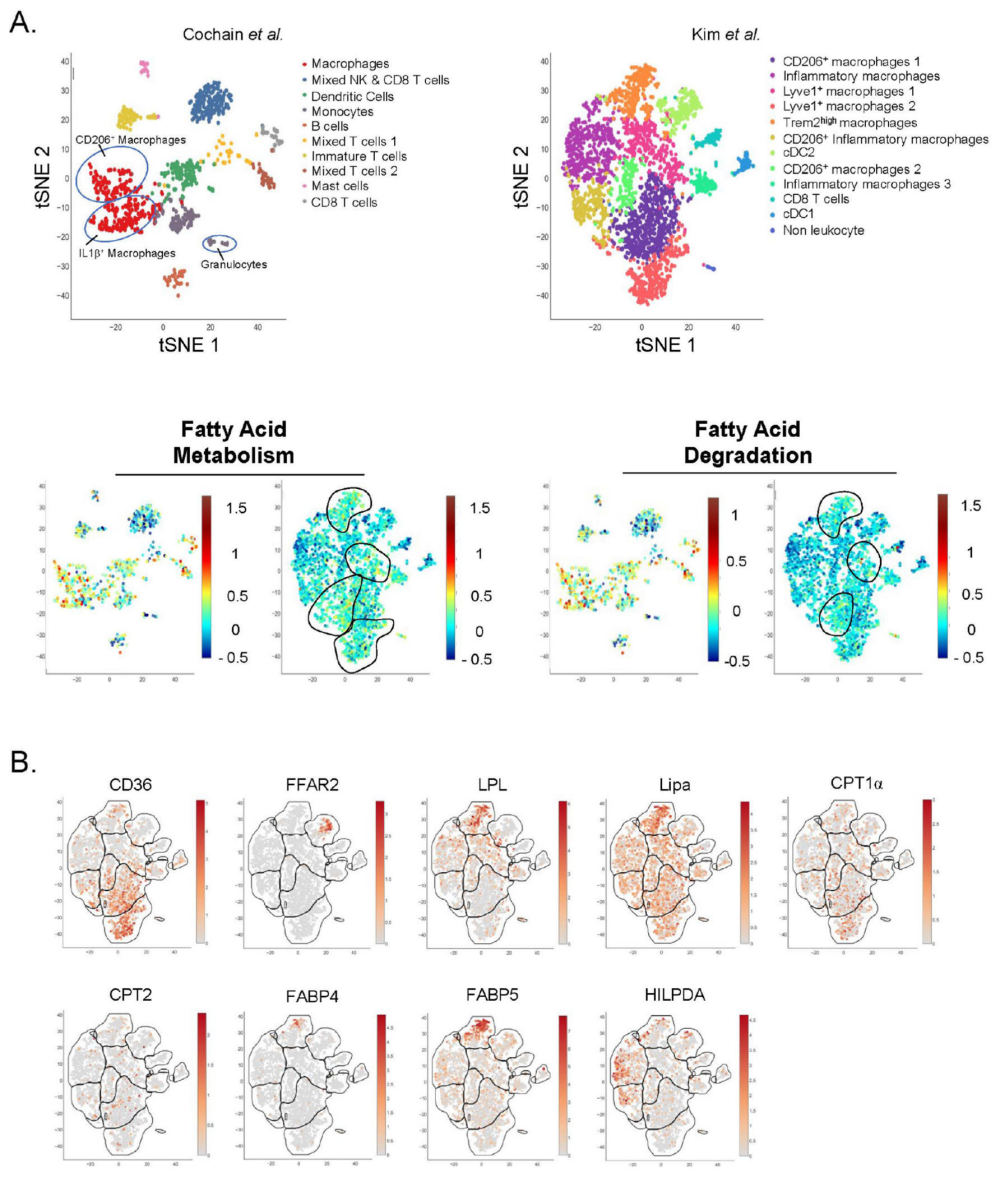
Single-Cell analysis of plaque immune cell lipid metabolism. (**A,B**) Single-Cell RNA-Seq of aortic CD45^+^ cells from Ldlr^−/−^ mice fed a HFD for (left) 11 weeks or (right) 12 weeks. Data from (left) Cochain et al. [21] (GSE97310) and (right) Kim et al. [23] (GSM3215435) were analyzed using the Single-Cell Explorer software (Artyomov lab). (**A**) Leukocyte clusters and corresponding KEGG Metabolic Pathway analysis. Fatty-Acid metabolism: KEGG mmu01212. Fatty-Acid degradation: KEGG mmu00071. **List of markers used in Cochain et al**.: Macrophages (mixed subsets): Itgam, Fcgr1, MerTK. Mixed NK and CD8 T cells: CD3, CD8, Klrb1c, Ncr1, Gzmb. Dendritic Cells (mixed subsets): Itgax, Ciita, Zbtb46. Monocytes: Itgam, Fcgr1, Ly6C, CCR2. B cells: Ciita, CD19, CD79α/β. Mixed T cells: Lck, CD3, CD4, CD8, Rag1. Immature T cells: Lck (−), CD3(+), Rag1 (−), CD4 (−), CD8 (−). Mast cells: Furin, Il1rl1. CD8 T cells: Lck, CD3, CD8. **Markers used in Kim et al. are in the legend for** Figure 1A. (**B**) Expression pattern of genes involved in lipid metabolism (Kim et al. [23] (GSM3215435)).

Emerging immunometabolism-centered studies have mainly focused on macrophages, while only a few studies investigated monocyte metabolic requirements. This may partly be explained by technical difficulty of using undifferentiated monocytes in *in vitro* cultures. Recent studies pointed towards lipid metabolism as an important factor of monocyte homeostasis. Using a BM transplant approach, Babaev and colleagues reported a decreased CCR2 expression on blood monocytes from Ldlr^−/−^ mice with FABP5^−/−^ (Fatty-acid binding protein) hematopoietic compartment, suggesting a chemotaxis-dependent proatherogenic role of myeloid FABP5 expression [123]. FABPs regulate intracellular lipid traffic and control their access to specific organelles. By contrast, FABP4 deletion in immune cells had no impact on plaque development [123]. ApoE^−/−^ mice with myeloid-specific deletion of lipoprotein lipase (Lpl) displayed decreased plaque development [124]. Lpl hydrolyzes circulating TGs and control their levels. Impaired monocyte generation and differentiation to macrophages were observed in a mouse model of Lpl deficiency and were attributed to Lpl-dependent regulation of CSF1R signaling [125]. Interestingly, FABP5 and Lpl mRNA were highly and selectively expressed in Trem2^+^ foam cells (Figure 2A,B). This could suggest that the atheroprotective effects of myeloid-specific deletion of Lpl might not be caused solely by monocytes but could additionally be the consequence of foam cell dysfunction.

The importance of monocyte lipid metabolism was challenged by recent data. Jordan and colleagues observed a decrease in blood monocyte numbers during fasting in humans. This effect could not be reverted by fat supplementation in mice, while carbohydrate and protein supplementation both restored blood monocyte counts [126]. However, this modulation of monocyte counts was attributed to modulations of CCL2 production through a liver-BM axis, and not to monocyte cell-intrinsic mechanisms. Nevertheless, monocytes from fasted mice were in a pronounced quiescent metabolic state in comparison to controls, as extracellular flux analysis of these cells showed reduction in both oxygen consumption rate and extracellular acidification rate. This was associated with up-regulation of inositol triphosphate metabolism and suppression of serine and glutathione metabolism. Overall, this metabolic adaptation of monocytes to fasting was associated with improved outcomes in models of chronic inflammatory diseases [126].

As atherosclerosis is associated with numerous systemic metabolic alterations, these exciting results support the urgent need to identify the dietary-related metabolic mechanisms controlling monocyte inflammatory and migratory potentials in this disease. Indeed, both qualitative and quantitative diet modulations could then be envisioned as non-invasive prophylactic therapies for patients presenting monocytosis and metabolic syndrome.

#### Macrophages

Lipid-laden macrophages were first described inside of atherosclerotic lesions in the late 1970s [127]. These macrophages, called foam cells, take up excessive cholesterol and oxidized LDL (oxLDL) particles via their scavenger receptors, which leads to the intracellular formation of lipid droplets [128–131]. The presence of these cells is considered as a hallmark of atherosclerosis, and foamy macrophages have historically been held culprit for plaque progression.

Intracellular accumulation of cholesterol has been linked to foam cell formation, cytokine production and atherosclerosis progression in a mouse model of defective cholesterol efflux [132]. Atherosclerosis progression has been associated with the formation of cholesterol crystals, which results from reduced esterification of free cholesterol [133]. These crystals can trigger inflammation through the activation of the NLRP3 inflammasome and subsequent IL-1β maturation, which have lately been a major focus of atherosclerosis research [134]. These results support a beneficial role for cholesterol efflux, which is mediated through the liver X receptor (LXR)-regulated transcriptional control of among others the cholesterol transporters ABCA1 and ABCG1. Mice deficient for LXRα and LXRβ display the formation of atheromatous lesions containing foam cells even in the absence of diet-induced hypercholesterolemia [135]. Subsequently, attempts have been made to decrease intracellular cholesterol accumulation and foam cell formation by the use of synthetic LXR agonists which have proven to be beneficial in pre-clinical models of atherosclerosis [136–138]. Consistently, myeloid-specific deletion of the LXR-regulated ABCA1/G1 cholesterol transporters was shown to exacerbate atherosclerosis development [139]. Alternatively activated (M2) human monocytes and macrophages were reported to be less responsive to LXR agonists [140]. Interestingly, they also displayed less foam cell traits despite decreased ABCA1 expression. This was countered by an improved cholesterol esterification capacity (an anti-inflammatory mechanism) [141], suggesting that efficient cholesterol handling may be more valuable than cholesterol efflux [140]. PPARα stimulation positively regulates ABCA1 expression [142]. Additionally, PPARγ activation also increases ABCA1 expression via LXR [143]. PPARα prevents foam cell formation and decreases plaque development [144,145]. PPARγ agonist also decreased foam cell formation [144]. The role of PPARs in macrophage biology and atherosclerosis is extensively reviewed in [146,147]. However, whether and how precisely PPARs affect specifically different myeloid cell populations in plaque remains to be defined. We recently reported that lysosomal acid lipase (LIPA)-dependent cholesterol hydrolysis promotes macrophage efferocytic capacity [148]. LIPA is expressed by all plaque macrophage subsets and is particularly enriched in foam cells (Figure 2B). The relevance of our observations linking LIPA activity and efferocytosis to atherosclerosis needs to be further investigated, as multiple studies reported a correlation between LIPA variants and coronary artery disease [149,150].

Macrophage foam cell formation is regulated by natural antibodies recognizing modified LDL particles and apolipoproteins. Indeed, the inhibition of oxLDL uptake by oxLDL-specific natural IgMs, which mask oxidized epitopes, decreases foam cell formation, inflammation and atherosclerosis development [151–153]. The production of oxLDL-specific antibodies, both of the IgM and IgG isotype, occurs during the development of atherosclerosis [154]. IgG-containing immune complexes are recognized by FcγR receptors, and ApoE^−/−^ mice deficient for FcγRIIb/CD32b, a low affinity inhibitory receptor, showed reduced plaque lipid content suggesting lower foam cell formation [155]. This was associated with an overall increase in plaque stability, as well as in circulating levels of oxLDL and IgG-ApoB immune complexes, suggesting a lower uptake of these particles by macrophages [155]. Foam cell formation was also reported to be induced by ApoA1-specific IgG in vitro [156]. Although atherosclerosis-associated IgMs and IgGs display different roles in foam cell formation, only the former are robustly associated with a (protective) role in the development of the disease [157].

In macrophages, a consequence of foam cell formation could be lipid overload-induced toxicity, which may hypothetically be a determinant of plaque necrosis. Both fatty acid metabolism and degradation pathways are enriched in plaque macrophages (Figure 2A). The hypoxia-inducible lipid-droplet-associated (Hilpda) protein has emerged as a key player in lipid droplet handling. Its role as a lipid-sensor and inhibitor of ATGL (the rate limiting enzyme of adipose tissue lipolysis) promotes lipid accumulation into lipid droplets and foam cell formation [158]. In plaques, Hilpda mRNA was preferentially expressed in inflammatory macrophages (Figure 2B). This Hilpda-mediated lipid storage was reported to be necessary for the maintenance of macrophage viability upon lipid overload, suggesting a beneficial role for lipid storage in terms of survival [159]. Nevertheless, Hilpda deficiency was shown to decrease atherosclerosis development and plaque lipid content, without affecting plaque macrophage apoptosis [159]. The authors attributed this phenotype to the Hilpda-dependent macrophage lipid accumulation and production of prostaglandin E2, which promotes vascular inflammation [160].

Overall, previous reports point towards a proatherogenic role for foam cells. However, the emergence of omics approaches led recent studies to challenge this view. Foamy peritoneal macrophages extracted from Ldlr^−/−^ mice fed a high cholesterol diet surprisingly showed a LXR-mediated down-regulation of genes associated with inflammatory responses and chemotaxis [161]. A turning point in foam cell research was reached by Kim and colleagues, who developed a strategy to isolate and characterize plaque foam cells using a lipid probe-based strategy [23]. Surprisingly, their results showed that foam cells only represent around 10% of aortic macrophages in atherosclerotic mice, although this proportion may vary depending on isolation efficiency. Moreover, both bulk and single cell RNA-Seq approaches showed that foamy macrophages are rather anti-inflammatory in comparison to non-foamy plaque macrophages. As discussed earlier, this is of particular interest in the context of the recent CANTOS trial, as NLRP3 and IL-1β expression were clearly a feature of non-foamy macrophages [23]. While previous studies relied greatly on in vitro models to analyze macrophage lipid metabolism and foam cell formation, this innovative approach may have supplied the methodology to further characterize foam cells in vivo. As discussed earlier, studying macrophages in the context of their micro-environment has repeatedly proven to be the key to understanding their biology.

In vitro studies revealed that IL-4 induces a specific metabolic profile in macrophages. These cells are named alternatively activated (M2) macrophages and rely on fatty acid oxidation for their metabolic needs [162]. Seminal studies demonstrated that fatty acid oxidation inhibition in macrophages prevents their M2 phenotype. This concept was recently challenged by the demonstration that etomoxir, a specific Cpt1a inhibitor, has “off-target” effects even at fairly low concentrations [163,164]. Genetic Cpt2-deletion failed to affect macrophage alternative polarization, further challenging the previously established dogma [165]. Cpt1 and Cpt2 mRNA were detected in plaque-resident myeloid cells without a subset-specific signature (Figure 2B). Whether plaque resident macrophages rely on fatty oxidation and Cpt1 remains currently unknown. Importantly, macrophage alternative activation depends on CD36, a membrane receptor for long chain fatty acids [162]. Single-cell RNA-Seq analysis revealed that CD36 is highly expressed in Lyve1^+^ plaque resident macrophages (Figure 2B). Of interest, Lyve1 is a canonical M2 activation marker which, together with CD36 expression, might help identifying the real in vivo alternatively activated macrophage relying on fatty acid oxidation (at least in the context of the plaque). CD36 is involved in non-classical monocyte patrolling during atherosclerosis induction [166]. Previous reports demonstrated that CD36 plays a crucial role during atherosclerosis development but it remains unclear how CD36 governs plaque myeloid cell metabolism [167–170]. For instance, CD36 signaling is involved in ROS generation and controls macrophage cytoskeleton organization [171].

#### Dendritic cells

One of the major proofs of DCs implication in atherosclerosis development is their impact on cholesterol homeostasis. Indeed, DC depletion in hyperlipidemic CD11c-DTR ApoE^−/−^ mice leads to increased hypercholesterolemia but no change in atherosclerosis due to lower DC-driven T-cell activation, suggesting that there is a close relationship between DCs and cholesterol homeostasis. CD11c expression in plaque is not restricted to DCs, and this function might be shared with CD11c-expressing macrophages (Figure 1B). Increasing DC survival by overexpressing Bcl2 leads to decreased cholesterol plasma levels [172]. However, long term DC depletion led to a progressive myeloproliferative syndrome, highlighting an indirect impact on the hematopoietic system [173]. Additionally, in Ldlr-deficient mice, DCs in atherosclerotic lesions have been shown to capture oxLDL contributing to foam cell formation and therefore to atherosclerosis progression [174].

Despite the fact that pDCs are present in a relatively low frequency in human and mouse atherosclerotic plaques, this cell type also plays a role in atherosclerosis development. pDCs numbers are increased in aortas of ApoE^−/−^ and Ldlr^−/−^ mice fed a high-fat diet [24,175]. Intriguingly, ApoE^−/−^ mice depleted in pDCs display decreased lipid-containing area, lower T cell activation and lower macrophage accumulation in the plaque [176]. In addition, when treated with oxLDL, pDCs show increased phagocytic capacity as well as a stimulated antigen-specific T cell response [177]. Genetic pDC depletion, following diptheria toxin administraton in BDCA2-DTR atherogenic mice, led to increased lesion area [175]. Moreover, TLR-induced IFN-I production by pDCs is triggered by neutrophils NETs in human atherosclerotic plaque [178]. All together, these data suggest that pDCs might be interesting targets in controlling the evolution of atherosclerosis. However, pDCs role in atherosclerosis is still under debate due to the opposite effects the antibody used against pDC bone marrow stromal cell antigen-2/PDCA1 has on Ldlr^−/−^ and ApoE^−/−^ mouse [176,177,179].

#### Neutrophils

Cholesterol metabolism appears to play a key role in neutrophil biology, as both Ldlr^−/−^ and ApoE^−/−^ mice fed a high fat diet display increased blood neutrophil numbers [119]. Cholesterol efflux receptors such as ABCA1/ABCG1 notably regulate neutrophil adhesion and activation [180]. Moreover, neutrophil accumulation and NETosis have also been found in the context of defective cholesterol efflux induced by ABCA1 and ABCG1 deficiency [181] (for review see [182]). Additionally, inhibition of cholesterol efflux in myeloid progenitors led to increased neutrophil production while a disruption in the chemotactic axis CXCL12-CXCR4 in the BM led to neutrophilia and therefore amplified lesion formation [97]. Moreover, mice fed a high-fat diet show significant increase in circulating neutrophil numbers [183]. However, surprisingly, epidemiological studies in humans have shown a positive correlation between elevated numbers of circulating neutrophils and cardiovascular events, independently from serum cholesterol levels [184]. In addition, fatty acids have also been proposed to be involved in neutrophils metabolic demands. Indeed, fatty acid receptors including free fatty acid receptor-1 (FFAR1/GPR40), free fatty acid receptor 2 (FFAR2/GPR43), and GPR84 are expressed on neutrophils [185]. However, short term fasting in humans had no effect on circulating neutrophil levels [126]. Cell-autonomous effects of lipids on neutrophils and their relevance in atherosclerosis require further investigations.

### Glucose Metabolism in Myeloid Cells

#### Monocytes

Unlike tissue-resident immune cells, monocytes need to quickly adapt to their new environment after blood vessel extravasation and entry into peripheral tissues. This seems critical in atherogenic conditions, as recent evidence suggests that monocytes might contribute to the onset of the disease due to their sensitivity to the plaque micro-environment, rather than to a preexisting inflammatory phenotype. Notably, Williams and colleagues showed that newly-recruited monocytes lose motility as they differentiate into macrophages within the plaque, thus reducing their capacity to reach apoptotic cells located deeper within the plaque and perform efferocytosis [186]. This rapid adaptation probably requires an adjustment of metabolic pathways to the locally available substrates.

In humans, glucose metabolism disorders such as *diabetes mellitus* have been associated with cardiovascular diseases, though the underlying cellular mechanisms remain unclear [187,188]. The use of the glucose analog 18F-FDG in PET-CT imaging has brought to light a correlation between acute coronary syndrome and 18F-FDG accumulation (representative of glucose avidity) in the bone marrow and the spleen (the later probably reflecting extramedullary hematopoiesis) in at least two independent cohorts [189]. Interestingly, Oburoglu and colleagues showed that in vitro (human CD34^+^ cells) and in vivo (newborn mice), administration of 2-deoxyglucose, a partially non metabolizable glucose analog used to inhibit glycolysis, restricted myeloid differentiation while promoting erythroid differentiation of HSCs [190]. Consistently, using chimeric pre-clinical models of atherosclerosis, our group previously reported a decrease in myelopoiesis and plaque development in mice with partial deficiency for Glut1, the main glucose transporter in the hematopoietic compartment [191]. Increased glucose levels in diabetic mice drive myelopoiesis, further supporting the evidence that glucose metabolism favors myeloid cells generation [192]. Interestingly, Jordan and colleagues reported a direct relation between food intake and systemic CCL2 levels, which allows for monocyte egress from the bone marrow compartment to the blood circulation [126]. This effect was mainly attributed to glucose metabolism, as the authors observed a positive correlation between blood monocyte counts and the quantity of gavage-administered glucose. Furthermore, monocyte mobilization could be inhibited by gavage with 2-deoxyglucose [126]. As discussed earlier, the CCL2-CCR2 chemotactic axis governs monocyte recruitment to atherosclerotic plaques and progression of the diseases [47]. Importantly, monocyte CCR2 expression strongly associates with vascular wall inflammation in patients with CVD risk [193]. However, whether glucose affects chemokine receptor expression on monocytes and facilitates their entry into inflamed plaques remains to be explored. In a pre-clinical plaque regression model, it was demonstrated that lowering plasma glucose concentration prevents monocyte entry into the inflamed plaque and improves pathology resolution [192]. Nevertheless, whether glucose lowering therapies affect monocyte CCR2 expression and their ability to enter into plaques and differentiate into macrophages also requires further investigations.

#### Macrophages

**Figure 3:**
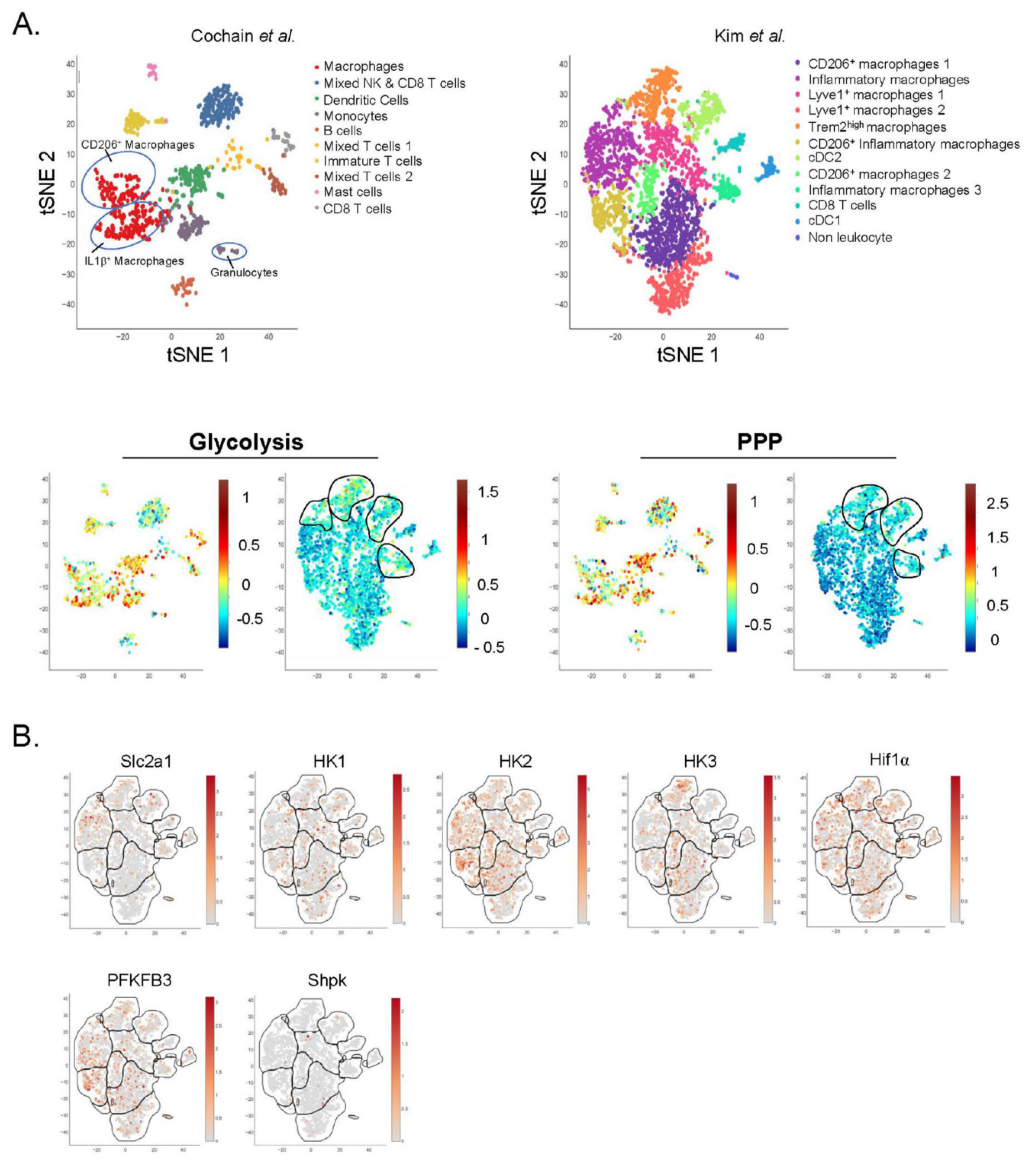
Single-Cell analysis of plaque immune cell glucose metabolism. (**A,B**) Single-Cell RNA-Seq of aortic CD45^+^ cells from Ldlr^−/−^ mice fed a HFD for (left) 11 weeks or (right) 12 weeks. Data from (left) Cochain et al. [21] (GSE97310) and (right) Kim et al. [23] (GSM3215435) were analyzed using the Single-Cell Explorer software (Artyomov lab). (**A**) Leukocyte clusters and corresponding KEGG Metabolic Pathway analysis. Glycolysis: KEGG mmu00010. Pentose Phosphate Pathway: KEGG mmu00030. The lists of markers used to identify subsets are in the legend of Figures 1 and 2. (**B**) Expression pattern of genes involved in glucose metabolism (Kim et al. [23] (GSM3215435)).

Macrophages rely on the membrane transporter Glut1, encoded by Slc2a1, for glucose entry. Slc2a1 is ubiquitously expressed among plaque myeloid cells (Figure 3A,B). However, transcriptomic analysis revealed an enrichment in transcripts related to glycolysis and PPP pathways in macrophages and DCs (Figure 3A,B). Glut1 is solely responsible for glucose entry into macrophages, as its ablation using genetic models demonstrated that other members of this family of transporters cannot substitute its absence [194]. Thus, Lyz2^cre^ × Slc2a1^fl/fl^ animals have minimal glucose entry associated with decreased levels of many glycolysis and PPP-related metabolites [194]. Compensatory mechanisms led to increased tricarboxylic acid cycle (TCA) metabolites in Glut1-deficent macrophages in comparison to controls [194]. Interestingly, when crossed to atherogenic Ldlr^−/−^ mice, Lyz2^cre^ × Slc2a1^fl/fl^ × Ldlr^−/−^ animals had similar plaque size as control mice [194]. Macrophage content, quantified by MOMA2 staining, remained similar as well. However, mice with Glut1-deficient myeloid cells had an elevated frequency of necrotic core per plaque that paired with a partial deficiency in efferocytosis [194]. This observation was confirmed in another study using the same genetic model [195]. Indeed, efferocytosis triggers a specific metabolic reprogramming of macrophages that relies mainly on glycolysis [195] and lowering glucose concentration, or pharmacological or genetic Glut1-inhibition all efficiently alter macrophage efferocytosis [195]. Glut1 expression is increased following macrophage TLR4 stimulation with LPS to facilitate glucose entry [196], though the relevance of this observation for plaque formation or maintenance requires further investigation. LPS also leads to accelerated glucose flux and increased glycolysis and PPP activation. This is supported by the transcriptional regulation of key enzymes involved in the aforementioned pathways. Thus, LPS increases the expression of two critical enzymes (HK3 and PFKFB3) involved in glycolysis, and this is paralleled by increased pro-inflammatory cytokine production [13]. In plaques, HK3 is found mainly in a population of Trem2^+^ macrophages, while PFKFB3 expression is higher in inflammatory macrophages (Figure 3B). Four HK (hexokinase) isoforms have been identified. Interestingly, HK1 was not enriched in a specific plaque immune subset, while HK2 is highly expressed in inflammatory macrophages (Figure 3B). Again, the biological significance of this observation needs further work. However, macrophage-specific Glut1 overexpression, despite increasing glucose entry and metabolization, failed to generate an increased pro-inflammatory cytokine production [13]. Plaque size, macrophage content and necrotic core area were similar between control and macrophage-Glut1 overexpressing animals [13]. This observation is surprising since increased glucose levels in mice have been associated with a macrophage pro-inflammatory phenotype and disease severity. Taken together, these observations suggest that glucose flux through Glut1 contributes to myeloid cells activation during atherosclerosis, but this is not sufficient to fully explain the pro-inflammatory phenotype of plaque macrophages. Macrophage alternative polarization also requires efficient glucose metabolism [197]. Blocking pyruvate mitochondrial entry and subsequent TCA incorporation leads to decreased ATP production [197]. This is consistent with the role of glucose in TCA cycle activation and ATP generation. Inhibition of the enzyme Acly, playing a key role in Acetyl-CoA generation, prevents macrophage alternative polarization in murine macrophages [198]. The human relevance of this observation was challenged in a recent report using pharmacological inhibitors and genetic approaches [199]. However, whether those pathways affect particularly the metabolic rewiring of a specific subset of plaque resident myeloid cells remains to be defined.

Interestingly, Folco and colleagues reported no modulations in glucose uptake when stimulating human primary macrophages with pro-inflammatory cytokines [200]. However, glucose uptake was increased in hypoxic conditions, along with increased HK2 expression, while HK1 expression remained unchanged. Immuno-histochemical analysis of human atherosclerotic lesions showed a colocalization of HK2 with the transcription factor HIF-1α (Hypoxia-inducible factor-1), a well-established regulator of glycolysis [200]. Hif-1α mRNA is ubiquitously expressed among plaque resident immune cells, most of which also express HK2 but not HK1, thus supporting these observations (Figure 3B). Advanced plaques contain hypoxic regions due to restricted blood supply and Hif-1a expression was detected in mouse and human plaques [201–204]. Conditional deletion of Hif-1α in myeloid cells (Lyz2^cre^ × Hif-1α^fl/fl^ mice) didn’t impact plaque size [205]. However, when Hif-1α was deleted in CD11c-expressing cells, an increased plaque area was documented, suggesting that this transcription factor mainly operates in CD11c-positive cells that could be DCs or a subset of macrophages (Figure 1B) [205]. CD11c^cre^ × Hif-1α^fl/fl^ mice displayed increased necrotic core area that might result from defective glucose-driven efferocytosis [205]. However, a recent report demonstrated that Lyz2^cre^ × Hif-1α^fl/fl^ mice have less plaque lesions when compared to control mice [206]. Surprisingly, Hif-1α deficient mice displayed less apoptotic cells and blunted glucose uptake [206].

LPS also regulates glucose flux into the PPP. LPS decreased the expression of the enzyme Shpk (CARKL) involved in the non-oxidative branch of the PPP [207]. Conversely, IL-4 induced CARKL expression is required for optimal macrophage alternative polarization. CARKL genetic deficiency forces glucose flux into the glycolysis pathway at a level similar to the one seen upon LPS challenge [207]. Shpk expression is not restricted to a selective myeloid cell population in plaques (Figure 3B). In advanced plaque, macrophage local proliferation contributes to plaque growth [208] and one would expect that the PPP pathway, involved in nucleotide generation, is highly activated. This was not yet documented to our knowledge. Interestingly, a recent report demonstrated that hypercholesterolemia suppressed the PPP in macrophages [209]. Whether this mechanism occurs in plaque during atherosclerosis development remains to be tested.

#### Dendritic cells

Several populations of DCs have been identified in plaques, both in mice and humans (Table 2 and Figure 1). However, little is known about the metabolic configuration of plaque resident DCs. Following TLR4 activation with LPS, DCs rapidly undergo a metabolic switch towards glycolysis [210,211]. LPS exposure increases glucose consumption rate and increases Glut1 expression in DCs [210]. This is paralleled by augmented nitric oxide (NO) production that subsequently decreases oxidative phosphorylation (OXPHOS) activity, ATP levels and mitochondrial activity. Consistently, activated DCs show less oxygen consumption rate than resting DCs [210]. Thus, NO seems to play a pivotal role in metabolic regulation [211]. In DCs, endogenous nitric oxide production inhibits OXPHOS and commit those cells to glucose metabolism and aerobic glycolysis similar to the Warburg effect described in tumor cells [211]. Additionally, LPS-induced NO production contributes to DCs induced death following activation [211]. This glycolytic reprogramming that happens within minutes after TLR stimulation is called the “glycolytic burst” and leads to *de novo* fatty acid synthesis needed for inflammatory cytokine production [212]. In addition, glucose restriction decreases activated-DC maturation, life span and cytokine secretion.

As compared to the rapid increase in glucose flux, Glut1 upregulation in DCs takes hours to build up following TLR stimulation. Therefore, exogenous glucose internalization seems unlikely to be the source required during early DC activation. This lag was recently solved by Thwe and colleagues who showed that intracellular glycogen reserves fuel DCs metabolic demands during early DC activation and that glycogen metabolism is required by these cells to initiate proper immune effector responses [213]. Of note, high glucose concentration increased the oxLDL-uptake capacity of DCs and augmented their IL-6 and IL-12 secretion while decreasing their IL-10 production [214].

#### Neutrophils

Unlike macrophages and monocytes, little is known about neutrophil metabolic configuration in health and disease. This might be explained by the difficulty to analyze these cells ex vivo. Moreover, single-cell RNA-Seq analysis on neutrophils is rather difficult because these cells typically possess lower number of transcripts when compared to T cells and macrophages. Neutrophils have long been thought to mainly rely on glycolytic metabolism [215], but neutrophils are able to switch from glycolysis to different metabolic pathways such as OXPHOS [216,217]. Increased glycemia favors granulopoiesis and neutrophil release in the blood circulation [191,192]. How modulation of plasma glucose levels impacts on neutrophil chemotaxis, especially their recruitment, retention and survival in the atherosclerotic plaque, is another exciting question.

### Amino Acids

In addition to glucose and lipids, amino acids are a source of energy for immune cells. Amino acids are essential metabolites for protein synthesis that act as intermediates in metabolic pathways. Amino acids modulate immune cell functions such as activation, differentiation, proliferation, gene expression, redox status or cytokine production. However, the role of amino acids on immune cell functions during atherosclerosis remains poorly understood.

In vitro studies have demonstrated the impact of glutamine metabolism on macrophage polarization [218,219]. More recently, Tavakoli et al. highlighted increased glutamine accumulation in aortas obtained from Ldlr^−/−^ mice. In this study, the autoradiography shows a non-homogenous glutamine accumulation that diverges according to macrophage activation profile. The combination of 2-deoxyglucose and glutamine accumulation within the aorta could predict the dominant macrophage polarization profile within the plaque. Indeed, a greater accumulation of glutamine than 2-deoxyglucose supposes a dominant anti-inflammatory population while the opposite indicates a higher content of pro-inflammatory macrophages. This study is one of the first to suggest a role for glutamine on plaque macrophage functions [220].

Besides glutamine, arginine is a key metabolite in vascular function and tone due to its role in the production of nitric oxide (NO). Indeed, arginine is metabolized both by arginase 1 (Arg1) and inducible nitric oxide synthase (iNOS) to produce ornithine and urea or NO respectively. As those two enzymes compete for the same substrate, the use of arginine by Arg1 limits NO production, a macrophage pro-atherogenic factor [221]. In Ldlr^−/−^ mice, microarray analysis showed an increase Arg1 expression in carotid artery during early atherosclerotic lesions. Moreover, Arg1 deficiency promoted NO synthesis upon lipid loading. Hematopoietic Arg1 deletion induced increase foam cell formation in the peritoneum. However, after 10 weeks of western diet, Arg1 deficiency in Ldlr^−/−^ mice had no effect on the plaque size nor on the plaque composition [222]. Similarly, in Ldlr^−/−^ mice deficient for Arg1 specifically in myeloid cells, Yurdagul et al. did not observe any phenotypic difference within the plaque. However, in a regression model, the authors demonstrated defective efferocytosis within the plaque leading to impaired regression, increased necrotic core area and decreased cap thickness. In addition, ornithine produced by Arg1 can be subsequently metabolized to putrescine by ODC (ornithine decarboxylase) [223,224]. Putrescine supplementation improves plaque macrophage efferocytosis leading to reduced lesion and necrotic core size, as well as cap thickness [225]. Conversely, ApoE^−/−^ mice deficient for iNOS in the bone marrow compartment have reduced atheromatous lesions showing that leukocyte mediates the pro-atherogenic effect of iNOS in mice [226].

## Conclusions and Future Directions

The field of immunometabolism is a rapidly expanding one that provides new insights on the role of specific metabolites in immune cells during health and disease. Atherosclerosis is characterized by increased plasma glucose and cholesterol concentrations, and we only recently started to appreciate how precisely these two metabolites impact on plaque resident myeloid cell functions and on their generation from bone marrow-derived precursors. The precise circuits incorporating glucose in macrophages, monocytes, DCs and neutrophils remain to be fully understood. Eventually this might help to apprehend how metabolism supports key functions specific for each population. For example, understanding how metabolism guides monocyte recruitment to plaques as well as their retention or eventual egress will be of significant importance for the field. Regarding macrophages, we recently learned that glucose metabolism sustains one of their key functions: the removal of apoptotic cells [194,195]. Whether glucose modulates macrophage motility in plaque or their interaction with the extracellular matrix remains to be elucidated. Recent reports demonstrated that DCs cytokine production is tightly regulated by their metabolic configuration [227]. Glucose metabolism regulates DCs migration via regulation of the key chemokine receptor CCR7 [228,229]. Of note, the role of CCR7 during atherosclerosis remains debated with studies reporting that CCR7-deficient mice display smaller [230], similar [231] and increased [232] plaque area. One might wonder whether this mechanism occurs during atherosclerosis as well. Specific metabolic configuration might be required for efficient peptide presentation to conventional T cells via MHC II or lipids to NKT (Natural Killer T) cells via CD1d. Another crucial question is whether the way of metabolite incorporation in myeloid cells affects their intracellular distribution. Apoptotic cell ingestion by myeloid cells leads to internalization of metabolites contained in the dying cell [225]. Thus, the efferocytes need to either incorporate these metabolites into their circuits, store them in specialized compartments or expulse them in the interstitial space where they could be used by neighbor cells.

Single-Cell RNA-Seq moved the field forward toward a better understanding of the immune diversity and functions in atherosclerotic plaque. Nevertheless, the predictions generated via this technique need in detail in situ validation. Recently, an elegant approach was validated to investigate single-cell metabolism in local environment [233]. This new technical advance will be helpful to investigate whether different myeloid cell populations residing in plaque possess a unique enzymatic profile. These analyses could also reveal a zonation in plaque enzyme and metabolite distribution. The field will benefit from our future ability to dose locally metabolites at the scale of the milieu surrounding a cell as well as cellular micro-compartmentalization. This, together with our ability to measure enzymatic activities and at the same scale will certainly make our task of making sense of metabolism an easier one. Our computing ability to integrate all those parameters will also facilitate the large-scale understanding of deciphering how access and competition for nutrients shape immunity and to what extent this can be used as new therapeutic handles.

## Figures and Tables

**Table 1:** Recent single-cell based studies assessing plaque composition.

References	Samples
Kim K et al., 2018 [23]	LdlR^−/−^ mice; 12 weeks HFD; aortic CD45^+^ cells
Cochain C et al., 2018 [21]	LdlR^−/−^ mice; 12 weeks HFD; aortic CD45^+^ cells
Winkels H et al., 2018 [22]	LdlR^−/−^ mice; 12 weeks CD or HFD; aortic CD45^+^ cells
	Transcriptomic data; 126 samples from the biobank of Karolinska Endarterectomies
Fernandez DM et al., 2019 [20]	Endarterectomy plaque samples
Cole JE et al., 2018 [24]	ApoE^−/−^ mice; CD or HFD 12 weeks; aortic CD45^+^ cells
Kalluri AS et al., 2019 [25]	WT digested aorta

**Table 2:** Summary of the reported plaque leukocyte proportions across the reports mentioned in Table 1.

Refs	Samples	Method	Macrophages	Monocytes	Dendritic cells			Neutrophils	T cells		B cells	NK
					pDC	cDC1	cDC2		CD4^+^	CD8^+^		cells
[23]	Male LdlR^−/−^ HFD 12 weeks	Single cell RNA-Seq	83.9%*	ND	ND	2.2%*	6.9%*	ND	3%*		ND	ND
[21]	Male LdlR^−/−^ HFD 11 weeks	Single cell RNA-Seq	28.9%	12.3%	14.9%			2%	8.7%	19.6%	2%	4%
	Male LdlR^−/−^ HFD 20 weeks		49.6%	ND	14.2%			ND	8.5%	16.8%	2.1%*	2.4%*
[22]	Male LdlR^−/−^ CD	Single cell RNA-Seq	13.6%	Myeloid cells: 6.2%					54.1%		24.4%	1.7%
	Male LdlR^−/−^ HFD 12 weeks		27%	Myeloid cells: 21.1%					45.8%		4%	2.1%
	Female ApoE^−/−^ CD		4.9%	Myeloid cells: 10.3%					60.6%		21.9%	2.4%
	Female ApoE^−/−^ HFD 12 weeks		9.6%	Myeloid cells: 12.6%					49%		27.2%	1.6%
	Human	Bulk RNA-Seq deconvolution	50%*	12%	ND			ND	20%*		10%*	5%
[20]	Human	CyTOF	10.6%	2.5%	0.4%	0.1%	ND	0.1%	31.6%	31.1%	2.6%	4.1%
	Human	CITE-Seq	16%	ND	6%*	1.5%*	ND	ND	20%*	26%*	8% *	11%*
[24]	ApoE^−/−^ sex unspecified CD	CyTOF	60%	2.5%	0.25%	1.6%	8.5%	2.5%	3%	3%	8%	1%
	ApoE^−/−^ sex unspecified HFD 12 weeks		57%	7%	1%	1.8%	6.5%	4%	3%	3%	5%	0.75%
[25]	Female WT CD	Single cell RNA-Seq	23%*	73.4%*	3.3%*			ND	ND		ND	ND

When possible, we reported the proportion of each cell type among total leukocytes as indicated by the authors. Stars (*) indicate missing information that was estimated and completed using the Single-Cell Explorer software (Artyomov Lab, Washington University St Louis). WT = Wild-Type. CD = Chow Diet. HFD = High Fat Diet. ND = Not Determined.
